# Enabling and promoting walking rehabilitation by paired associative stimulation after incomplete paraplegia: a case report

**DOI:** 10.1038/s41394-020-0320-7

**Published:** 2020-08-13

**Authors:** A. Shulga, S. Savolainen, E. Kirveskari, J. P. Mäkelä

**Affiliations:** 1 BioMag Laboratory, HUS Diagnostic Center, Helsinki University Hospital, Helsinki, Finland; 2 Clinical Neurosciences, Neurology, Helsinki University Hospital and University of Helsinki, Helsinki, Finland; 3 HUS Medical Imaging Center, Clinical Neurophysiology; Clinical Neurosciences, Helsinki University Hospital and University of Helsinki, Helsinki, Finland

**Keywords:** Spinal cord diseases, Trauma, Spike-timing-dependent plasticity

## Abstract

**Introduction:**

Paired associative stimulation (PAS) is a combination of transcranial magnetic stimulation (TMS) and peripheral nerve stimulation (PNS) and induces plastic changes in the human corticospinal tract. We have previously shown that PAS consisting of TMS pulses given at 100% of stimulator output and high-frequency PNS is beneficial for motor rehabilitation of patients with a chronic incomplete spinal cord injury (SCI). The therapeutic possibilities of this PAS variant for walking rehabilitation of paraplegic patients are unexplored.

**Case presentation:**

A 47-year old man with traumatic incomplete paraplegia (AIS D, neurological level T7) received PAS to his left leg for 3 months at 12 months post injury (PAS1) and for an additional 3 months at 24 months post injury (PAS2). The right leg had normal AIS scores and was not stimulated. Before PAS, the patient was nonambulatory, could not stand without weight support, and was consequently not eligible for conventional walking rehabilitation. After PAS1, the patient could stand for 1.5 min and take 13 steps (24 steps in follow up) on parallel bars without weight support and was enrolled into conventional walking rehabilitation. He achieved independent walking ability with a rollator. During PAS2, walking distance increased 2.4 times faster than during the preceding year. The left leg AIS score and spinal cord independence measure mobility subscore increased. No adverse effects were detected.

**Discussion:**

This is the first report of PAS with a high-frequency peripheral component that enabled and promoted walking rehabilitation. Together with previous reports on this technique, this result encourages further research into its therapeutic potential and mechanism.

## Introduction

Paired associative stimulation (PAS) is a combination of non-invasive transcranial magnetic stimulation (TMS) with peripheral nerve stimulation (PNS) [1, 2]. Near-simultaneous TMS to primary motor cortex (M1) and PNS pulses to peripheral nerves are presumed to induce long-term potentiation (LTP)-like plasticity in the human corticospinal tract [3, 4]. Conjoint activation of two neuronal ensembles in PAS is thought to lead to a system-level response where neuronal excitability and connectivity are altered in a long-term way according to the Hebbian rule of associative plasticity [3]. The first PAS protocols targeted cortical connectivity, but synchronous activation of upper and lower motor neurons can also modify spinal excitability [3, 5]. PAS has been shown to benefit patients with incomplete spinal cord injury (SCI) [6, 7].

Since conventional PAS protocols strongly depend on the exact determination of the interval between TMS and PNS (interstimulus interval, ISI) and on numerous other conditions [4], we developed a modified version of PAS. This version utilizes high-frequency PNS trains and TMS pulses given at 100% of stimulator output and leads to a robust motor-evoked potential (MEP) potentiation, an indicator of LTP-like plasticity [8, 9]. Unlike conventional protocols, this modified PAS does not require exact calculation of ISI between TMS and PNS and is not highly sensitive to small inaccuracies in TMS target determination [8, 9]. Thus, it is feasible in challenging clinical conditions where measurements with millisecond precision are not always possible. A high-intensity TMS pulse is thought to generate multiple orthodromic volleys, whereas a peripheral stimulus train generates multiple antidromic activations in the corticospinal tract. Their multiple collisions at the level of the spinal cord are presumed to induce an LTP-like effect; cellular-level studies have shown that upon multiple interactions, LTP-like effects overcome their long-term depression-like counterparts [10].

We have reported altogether 18 patients with chronic incomplete SCI. The patients have benefited from this version of PAS and have gained increased independence and better motor function of upper or lower limbs [7, 11–14], including the clinically beneficial effect of PAS on lower limb function in incomplete tetraplegic patients [14]. No reports on the effect of PAS on walking of paraplegic patients exist. Here, we describe the results of PAS given to a patient with incomplete paraplegia in walking rehabilitation. We report for the first time a therapeutic contribution of PAS in regaining independent overground walking.

## Case presentation

The study was approved by the Ethics Committee of the Helsinki University Hospital. The patient provided written informed consent. A 47-year old male with an SCI was enrolled in the study at 12 months post injury. The patient had incomplete paraplegia (AIS D, neurological level T7) due to traumatic fracture of T9-T10 vertebrae. The patient could move both lower limbs but was nonambulatory (Supplementary Video 1) and was not able to stand without support. After the initial standard rehabilitation at the acute and subacute stages, the patient was not referred to further walking rehabilitation since achievement of overground walking was not deemed realistic.

The patient received an initial 3-month period of paired associative stimulation (PAS 1) at 12 months post injury and a second 3-month period at 24 months post injury (PAS 2). The AIS score of the right leg was normal (25 points) at onset; therefore, we only stimulated the left leg.

PAS consisted of navigated transcranial magnetic stimulation (nTMS; eXimia magnetic stimulator, Nexstim Ltd., Helsinki, Finland) combined to electrical stimulation trains to peripheral nerves (PNS, administered with Dantec Keypoint device, Natus Medical Inc., Pleasanton, CA, and surface electrodes, Neuroline 720, AMBU A/S, Ballerup, Denmark).

For TMS, we defined hotspots in the primary motor cortex (M1) for abductor hallucis (AH), extensor digitorum brevis (EDB), vastus medialis (VM), and gluteus maximus (GM) muscles [14]. Mapping at the suprathreshold intensity was started at the presumed anatomical location of the representation of these muscles. The location and direction of the coil was thereafter varied to define the sites (hotpots) where TMS elicited the largest and the most consistent motor-evoked potentials (MEPs) recorded with the surface electrodes placed on the corresponding muscle belly. The resting motor threshold (RMT) in all muscles was over 100% of the maximum stimulator output (MSO) of the TMS device and therefore all M1 mapping was performed with a weak motor preactivation. TMS was delivered at 100% of MSO during PAS, as described previously [9, 12].

TMS of AH hotspot was paired with PNS of the tibial nerve, EDB with peroneal nerve, VM with femoral nerve, and GM with gluteal nerve according to the innervation of these muscles (Table 1) to obtain coverage of all major muscle groups of the lower limb. PNS consisted of trains of six 1-ms pulses delivered at 100 Hz [12–14]. For the gluteal nerve stimulation, the electrode placement was determined by an anatomical landmark centered at the ischial tuberosity [14]; a tape roll (45 × 25 mm) was attached on top of the electrodes and the patient sat on it, thus pressing the electrodes toward the nerve (see [14]). For the femoral nerve stimulation, the electrodes were placed at the crossing of the inguinal crease and femoral artery; the electrodes were slightly pressed manually to ensure that the stimulation reached the nerve. The contraction of the VM muscle during femoral nerve stimulation was monitored and the optimal site of stimulation was adjusted to achieve maximal contraction. The tibial nerve was stimulated behind the medial malleolus and the peroneal nerve at the frontal midline of the ankle (see Fig. 3 in [7] for photos of tibial and peroneal nerve stimulation electrodes).

**Table 1:** TMS and PNS targets for PAS protocol covering the major muscle groups of the lower limb.

TMS	PNS	Muscle groups
Gluteus maximus hotspot	Gluteal nerve	Hip extensors
		Hip abductors
Vastus medialis hotspot	Femoral nerve	Hip flexors
		Knee extensors
Abductor hallucis hotspot	Tibial nerve	Knee flexors
		Ankle plantarflexors
Extensor digitorum brevis hotspot	Peroneal nerve	Ankle dorsiflexors

F-responses were recorded with a Dantec Keypoint electroneuromyography device and surface electrodes as mentioned above. F-waves are orthodromic responses produced by a pool of motoneurons which is antidromically activated upon PNS. F-waves thus reflect conduction to and from the spinal cord and were used for two purposes as described below: to calculate the ISI between TMS and PNS, and to determine PNS intensity. The tibial, peroneal, femoral, and gluteal nerves were stimulated at the sites described above. Ten responses to 0.2-ms pulses at supramaximal intensity were recorded from the same muscles as in MEP measurements to determine the minimum F latency for ISI calculation (see below). Thereafter, F-responses to single 1-ms pulses were recorded. The minimum intensity of PNS required to produce persistent F-responses with single 1-ms pulses defined PNS intensity for each nerve; this procedure makes sure that the stimulation of motoneurons reaches the spinal cord [11–13]. The resulting PNS intensities in PAS 1/PAS 2 were 30/50 mA for femoral, 85/66 mA for gluteal, and 40/17 mA for tibial nerves. Peroneal nerve F-responses could not be detected before PAS 1 and we used a 40-mA stimulation intensity as in the tibial nerve; before PAS 2, the responses were detected, and the intensity was 17 mA.

Each TMS pulse was synchronized with the first pulse of PNS train at a pre-calculated ISI. ISI was calculated by a formula (F-response latency minus MEP latency) to coincide the stimuli at the level of the spinal cord. This formula utilizes minimum F-latency determined as described above and mean latency of 10 MEPs recorded at 120% RMT (in this case, at 100% MSO). Please see [15] for a detailed description and rationale of the formula.

Paired stimulations were delivered at 0.2 Hz. In PAS 1, each nerve was stimulated for 30 min (360 stimulations); in PAS 2, the duration was 20 min (240 stimulations). The time required for preparations was ~30 min. The entire session (four nerves plus preparation) lasted around 2 h 30 min in PAS 1 and ~1 h 50 min in PAS 2. In both PAS 1 and PAS 2, we administered the stimulation 5 days per week during the first 2 weeks and 3 days per week thereafter. Since RMT was over 100%, the patient was instructed to slightly preactivate the muscles corresponding to each nerve during PAS (plantarflexion and knee flexion for PAS involving tibial nerve, 10 min + 10 min; dorsiflexion for peroneal nerve, 20 min; hip flexion and knee extension for femoral nerve, 10 min + 10 min; and gluteal muscle contraction and hip abduction for gluteal nerve, 10 + 10 min).

The patient listened to music of his own choice during a PAS session. The patient was seated in a comfortable armchair provided by the manufacturer of the TMS device in semiseated position. The therapist pressed the femoral nerve electrodes throughout femoral nerve stimulation as described above, manually monitored gluteal muscle contraction in the beginning of gluteal nerve stimulation over several pulses to ensure correct position of the electrodes (see above), and observed adequate muscle contraction during peroneal and tibial nerve stimulations. The therapist also reminded the patient of the required preactivation movements as described above, if needed, and monitored the correct position of the TMS coil with the navigation tool throughout the entire session. The skin below the electrodes was monitored after each session; slight redness of the skin after stimulation is attributed to increased blood flow and does not require further attention if reversible in within 2 to 3 h. No skin damage was observed.

The research team was not involved in or did not introduce any changes into medication or conventional physiotherapy of the patient. Standard conventional physiotherapy consisted of stretching tight/spastic muscles 1 h weekly and strengthening and standing exercises 1 h weekly. This routine remained the same before, during, and after both stimulation periods with one exception: during PAS 2, the patient had no physiotherapy during the first month of PAS and received physiotherapy only 1 h per week during the second and third months. During PAS 1, medication consisted of clonazepam 1 mg and tizanidine 2 mg daily. During PAS 2, tizanide was replaced by baclofen 5 mg 3 times daily.

An experienced physiotherapist evaluated AIS motor scores, walking, and modified Ashworth scale before, after, and 2 months after PAS 1 and PAS 2. A physician evaluated sensory scores before and after PAS 1 and after PAS 2. Walking distance was defined as the maximum distance the patient was able to walk with a rollator without significant discomfort or assistance. The time of the walking session was monitored and walking speed was calculated.

**Fig. 1:**
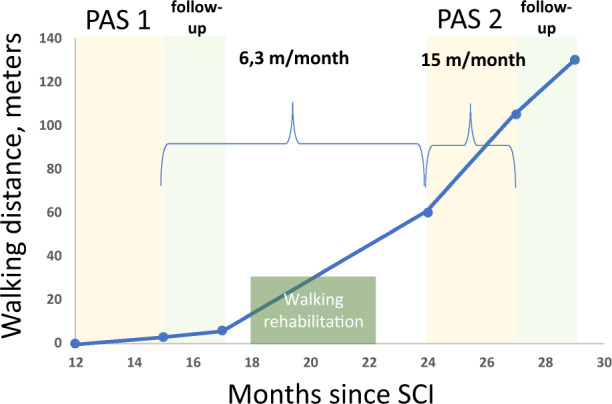
Patient’s maximum walking distance without assistance at different time points after SCI. PAS 1 and PAS 2 and their follow-up periods are shown. Intensive walking rehabilitation was administered for 4 months between PAS 1 and PAS 2 and was independent from the research project. Brackets show the extent of improvement prior to PAS 2 (6.3 m per month) and during PAS 2 (15 m per month).

**Fig. 2:**
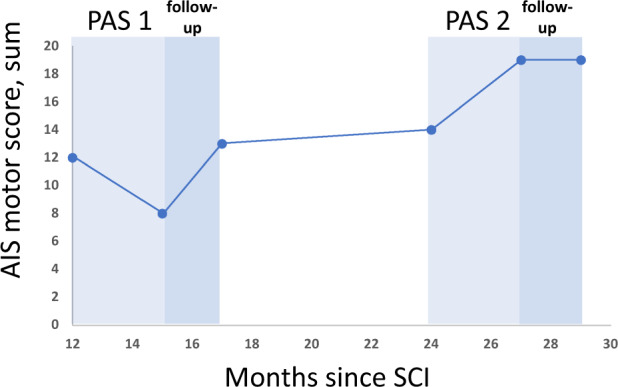
AIS motor score. Sum of the left (stimulated) leg AIS motor score at different time points after SCI is shown.

**Fig. 3:**
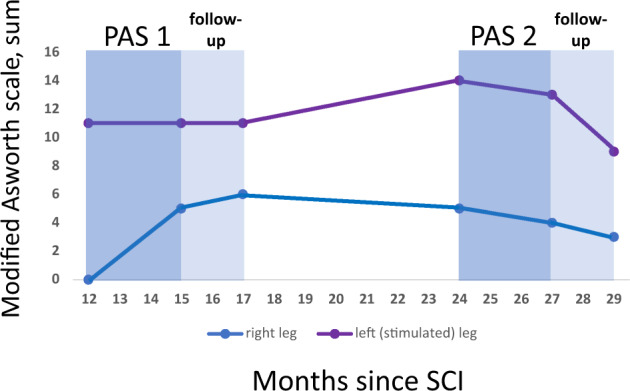
Spasticity evaluation. Sum of the modified Ashworth scale scores for the right (unstimulated) and left (stimulated) legs at different time points after SCI is shown.

Prior to PAS, the patient had muscle activity in both lower limbs but could not ambulate or stand without weight support (Supplementary Video 1). After PAS 1, the patient regained standing ability without support for 1.5 min (Supplementary Video 2) and could take 13 steps without weight support (Supplementary Video 3). During follow up, the patient could take 24 steps on parallel bars; the walking distance doubled (Fig. 1 and Supplementary Video 4). Paradoxically, AIS motor scores (Fig. 2) diminished immediately after PAS 1 (hip flexor and ankle plantar flexor score diminished by 2 points each, long toe extensors diminished by 1 point, knee extensors gained 1 point) but were restored to a level slightly above pre-PAS level after follow up (Fig. 2). This result is most likely explained by a technical error; AIS motor scores were measured before assessment of walking in all other evaluations except for post-PAS 1 evaluation, and low scores were most likely due to muscle tiredness after the walking measurement. Spasticity (Fig. 3) increased during PAS 1 and the follow-up period in the unstimulated (right) leg and remained at the same level in the stimulated (left) leg.

Before PAS 1, conventional walking rehabilitation was deemed futile. As the patient was able to stand and take steps without support after PAS 1, he was re-evaluated at the same conventional SCI clinic by a physician uninvolved in the research team and was enrolled into intensive walking rehabilitation for 4 months, which occurred between 18 and 22 months post injury (Fig. 1). PAS 2 started at 24 months post injury.

During the period between the end of PAS 1 and the beginning of PAS 2 (which included the intensive walking rehabilitation), the walking distance increased 6.3 meters per month (Fig. 1). Strikingly, during 3 months of PAS 2 (including very little conventional physiotherapy, see above), the walking distance increased 15 meters per month and continued increasing during the second follow-up period (Fig. 1).

AIS motor score remained stable during the period between PAS 1 and PAS 2 (Fig. 2). The motor score increased during PAS 2 and the achieved score persisted in the follow-up (Fig. 2).

Spasticity decreased slightly in both legs during PAS 2 (Fig. 3).

At PAS 2 follow up, the patient reported that he was walking with a rollator ~50% of the time and used a wheelchair 50% of the time when moving around the house. Walking speed was 0.17 m/s before PAS 2 and 0.22 m/s after PAS 2 and 0.21 m/s after PAS 2 follow up.

Mobility subscores of spinal cord independence measure (SCIM) improved after PAS 2 (Table 2). Scores for self-care (total score 18), respiration, and sphincter management (total score 33) did not change.

**Table 2:**
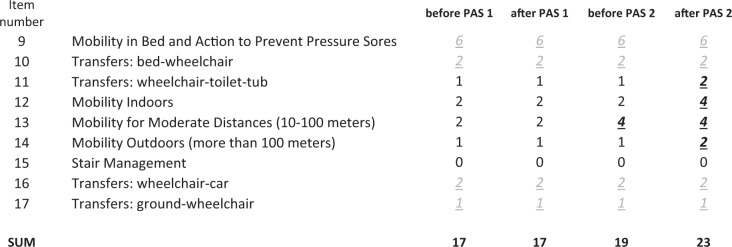
Spinal cord independence measure (SCIM), mobility subscore.

Normal scores at experiment onset are marked in gray italic underlined. Improved scores are marked in bold italic underlined.

The sensory scores did not change (Table 3). The effects on pain are reported in Table 3. After PAS 2, the patient subjectively reported better function of the bowel and subjectively perceived that the abdominal muscles were stronger; this was not measured objectively. No other effects on autonomic functions were reported.

**Table 3:** Sensory scores and pain.

	Right			Left (stimulated)		
	before PAS 1	after PAS 1	after PAS 2	before PAS 1	after PAS 1	after PAS 2
Sensory scores						
Light touch	4	6	7	2	2	2
Pin prick	1	1	1	0	0	0

Before PAS 1	After PAS 1	Before PAS 2	After PAS 2
Pain			
Unpleasant tingling in both legs (whole leg area) for about 5 h per day.	None	Continuous tingling and unpleasant sensations (grade 3–4 on visual analog [VAS] scale) in both legs starting from the mid-thighs and extending distally. Pain in lower abdomen.	Tingling and unpleasant sensations; similar as before PAS 2. Pain in lower abdomen was diminished.

MEPs were not measured post-PAS, since pre-PAS measurements were performed during weak preactivation (see above), which precludes objective evaluation of MEP amplitudes.

The patient did not experience any adverse effects during PAS 1 or PAS 2.

## Discussion

This is the first case report demonstrating that PAS with high-frequency PNS may contribute to regaining of overground walking after an incomplete paraplegia. We have previously focused on tetraplegic patients and demonstrated the benefits of PAS for upper [7, 11–13] and lower [14] extremity function. In our very first work [7] we showed restoration of ankle movements in a paraplegic patient by tibial and peroneal nerve PAS administered for 12 weeks. Here, we stimulated for the first time all four major nerves of the lower limb in a paraplegic patient in two periods of 12 weeks at two difference phases of rehabilitation; first when gaining sufficient strength to start walking rehabilitation, and then once overground walking was already achieved. As in our previous patient reports, the patient did not have a sports background and is representative of the usual population of SCI patients.

Since this is a case report, the exact role of PAS vs natural recovery and the impact of conventional rehabilitation naturally remains open. However, several points suggest that both PAS stimulation periods were beneficial. Previous research showed that in both incomplete paraplegia and tetraplegia, the majority of improvement occurred within the first 6–9 months and a plateau was reached by 12 months [16]. Here during PAS 1, the patient achieved independent standing and could take several steps without support even though the conventional physiotherapy was not modified. This was crucial for the patient to enter walking rehabilitation, which was considered unrealistic before PAS 1, since the patient was not able to stand or walk without considerable weight support (Supplementary Video 1). Although it is evident that in incomplete injuries some degree of improvement still occurs also after the first year, the observed major improvement suggests that PAS was a contributing factor. Together with our previous results [7, 11–14], this suggests that PAS might be useful for patients who require additional muscle strength needed for walking rehabilitation. PAS 2 was applied at the stage when the patient could already walk independently. It is noteworthy that the rate of change of walking distance was almost 2.5 times faster during PAS 2 (which included minimal conventional rehabilitation) than during intensive walking rehabilitation without PAS (Fig. 1). AIS motor score improved during 3 months of PAS 2, whereas it remained stable for 7 months before it (Fig. 2). SCIM score improved more during 3 months of PAS 2 than during the 9 months before it (Table 2).

Although no adverse effects were observed, the patient did perceive the procedure as time-consuming and somewhat tiring. However, the patient was able to continue his work and conventional rehabilitation during both PAS periods. Since only one leg was stimulated, we could stimulate all four major nerves (Table 1). In our recent work in tetraplegic patients where both lower limbs were stimulated [14], we selected the weakest nerves, limiting the number of stimulated nerves to six (and thus stimulation time to 2 h). We have recently shown in healthy subjects that administering the same amount of pulses but reducing the stimulation time to half results in weaker MEP potentiation and is therefore most probably not optimal for patients [17].

The treatment was a combination of TMS, PNS, and slight voluntary preactivation of the targeted muscles. When TMS alone is used, lasting inhibitory aftereffects can be achieved with 1-Hz repetitive TMS and facilitatory aftereffects with high-frequency (more than 1 Hz) repetitive TMS [18]. In healthy subjects, TMS and PNS components alone do not increase MEPs and thus do not induce plastic changes on their own [7, 9]. Thus, it is highly improbable that TMS alone would have accounted for the obtained results. We have also shown that PAS is more efficient than PNS in SCI patients [11]. A slight preactivation of each muscle group for 20 min three times per week is a minor addition to the patient’s other physical activity and cannot by itself explain the observed improvements.

Although the patient improved considerably, only a limited walking distance was achieved. At the end of the PAS 2 follow-up period, the patient still needed the rollator, which impairs the functionality and independence of walking. Nevertheless, the patient perceived the achieved improvement as valuable and worth the effort. Walking around the house 50% of the time benefits overall health e.g. by improving blood pressure regulation, bone density, weight control, bowel function, and psychological well-being, and prevents pressure sores and heterotopic ossification as compared to full inability to walk. Better results may occur if PAS is started at the subacute stage, before irreversible changes in muscle composition and neuronal rewiring have occurred.

Together with our previous studies [7, 11–14], this study justifies larger PAS trials for patients with different types and stages of SCI.

## Supplementary information

Supplementary Video legends

Supplementary Video1

Supplementary Video2

Supplementary Video3

Supplementary Video4

Supplementary Video5

Supplementary Information
