# Supplementary material for: Enabling and promoting walking rehabilitation by paired associative stimulation after incomplete paraplegia: a case report
Source: Spinal Cord Ser Cases. 2020 Aug 13;6:72. doi: 10.1038/s41394-020-0320-7 (PMC7426433; doi:10.1038/s41394-020-0320-7)
Supplement: Supplementary file 1 — Supplementary Video legends [file 41394_2020_320_MOESM1_ESM.docx]

**Supplementary Video legends**

**Supplementary Video 1:** Before PAS, the patient was able to ambulate only with weight support and Eva support walker.

**Supplementary Video 2:** After PAS 1, the patient was able to stand independently without weight support.

**Supplementary Video 3:** After PAS 1, the patient was able take several steps without weight support.

**Supplementary Video 4:** The achieved improvement shown in Supplementary Video 3 persisted and increased after 2 months of follow up.

**Supplementary Video 5:** Examples of overground walking before, during, and after PAS 2.
